# An outbreak of *Leishmania major* from an endemic to a non-endemic region posed a public health threat in Iraq from 2014-2017: Epidemiological, molecular and phylogenetic studies

**DOI:** 10.1371/journal.pntd.0006255

**Published:** 2018-03-01

**Authors:** Mariwan M. M. Al-Bajalan, Sirwan M. A. Al-Jaf, Sherko S. Niranji, Dler R. Abdulkareem, Khudhair K. Al-Kayali, Hirotomo Kato

**Affiliations:** 1 Department of Biology- College of Education and Research Centre, University of Garmian, Kalar- Sulaimaniyah, Iraq; 2 Department of Dermatology, Kalar General Hospital, Kalar- Sulaimaniyah, Iraq; 3 Department of Dermatology- College of Medicine, University of Diyala, Baquba- Diyala, Iraq; 4 Division of Medical Zoology, Department of Infection and Immunity, Jichi Medical University, Tochigi, Japan; Charité University Medicine Berlin, GERMANY

## Abstract

**Background:**

Cutaneous leishmaniasis (CL) is a neglected worldwide, zoonotic, vector-borne, tropical disease that is a threat to public health. This threat may spread from endemic to non-endemic areas. Current research has exploited epidemiological, molecular and phylogenetical studies to determine the danger of an outbreak of CL in the borderline area between northern and central Iraq from 2014–2017.

**Methodology/Principal findings:**

For the first time, using sequence analysis of the cytochrome *b* gene, the occurrence of CL in the borderline area between northern and central Iraq was confirmed to be due to *Leishmania major*. The phylogenetic analysis indicated that it was closely related to the *L*. *major* MRHO/IR/75/ER strain in Iran.

**Conclusions and significance:**

In conclusion, the genotype confirmation of the *L*. *major* strain will improve our understanding of the epidemiology of the disease. This is important for facilitating control programs to prevent the further spread of CL. Furthermore, this area could be considered as a model for further research on the risk of global CL epidemics in other non-endemic countries where both reservoir hosts and sandfly vectors are present.

## Author summary

Leishmaniasis refers to a disease with three main types of clinical manifestation in infected individuals including cutaneous, mucocutaneous and visceral forms. It is caused by several species of a parasite belonging to the genus *Leishmania* and is transmitted by a small blood-sucking insect called a sandfly. The disease is mostly confined to the majority of the poorest countries worldwide, including Iraq, and is categorized as a low priority public health concern. The risk of the disease is exacerbated especially when suitable environments assist the sandfly and reservoir host to breed and spread and help the parasite to transfer from high incidence areas to places free from the disease. Therefore, we investigated the risk of the CL form of the disease after an outbreak in a borderline between northern and central of Iraq using the most sensitive diagnostic techniques including PCR and gene sequencing. The epidemiological, molecular and phylogenetic analyses of the parasites were studied, and we found that the parasite species *Leishmania major* was associated with the outbreak. Phylogeny analysis confirmed that the identified strain of the parasite matched an Iranian strain. These results indicate the risk of the disease spreading from endemic to non-endemic areas.

## Introduction

Leishmaniasis is considered to be a neglected tropical and zoonotic disease that spreads via phlebotomine sandfly vectors [1]. Leishmaniasis is a parasitic disease caused by intracellular protozoa which in humans has four clinical forms including cutaneous (CL), diffuse cutaneous (DCL), visceral (VL) and mucocutaneous (MCL) leishmaniasis and it is endemic in different parts of the world [2]. The morbidity associated with human CL is up to 1.2 million cases distributed worldwide resulting in extensive integumentary lesions [3].

There are two groups of CL, New World and Old World leishmaniasis, with only the latter group identified in the Middle East and it includes three main species; *L*. *major*, *L*. *tropica* and *L*. *infantum* [4]. Recent studies showed a high prevalence of CL in Iran [5, 6], Turkey and Syria [7]. Although Iraq shares long borders with these countries and leishmaniasis is endemic, the World Health Organization has not classified it as a country with a high burden profile [8].

In Iraq, several studies have been performed to diagnose *Leishmania* parasites from skin lesions of human patients by using different methods including histopathological examinations, direct smears, cultures and serological tests [9, 10]. Few studies have been conducted to exploit PCR in the characterization of the *Leishmania* strains in human cutaneous lesions [11] and VL-suspected patients [12] in central Iraq. Studies have been performed without conducting gene sequencing or phylogenetic analyses. However, in a US military base in Southern Iraq, a phylogenetic study investigated the prevalence of different *Leishmania* species in sandflies using molecular study and phylogenetic analysis [13].

Therefore, the aim of this study was to identify the genotype of the most prevalent CL strains in the region using cytochrome *b* gene amplification by PCR and sequencing.

## Methods

### Geographical background of the study area

An outbreak of leishmaniasis was clinically suspected for the first time in 2013 in areas belonging to the Kifri district in the Garmian Administration. The term (Garmian) is a Kurdish word which is used to denote a ‘hot and dry area’ indicating information about location and climate. The Garmian area is located in the southeast Kurdistan region of Iraq. It is in between the latitudes (34°15–33 = - 35° 11–05 =) above the equator and the longitudes (44° 29–41 = - 45° 54–20 =) of the eastern hemisphere. The Garmian includes the districts: Kalar, Kifri, and Khanaqin, and its total area is 6731.73 square kilometers. According to the official site of the general board of tourism of Kurdistan- Iraq in 2015, the total population of the central town of Garmian, Kalar, is about 250,000 residents [14]. In this region, there is an increasing concern about the cutaneous form of leishmaniasis which is publicly known as “Baghdad sore”. Since 2014 leishmaniasis has been considered a notifiable disease and every new case with a clinical manifestation of cutaneous lesions of leishmaniasis should be recorded officially by local authority officers as a transmissible disease before the patient receives treatment.

**Fig 1:**
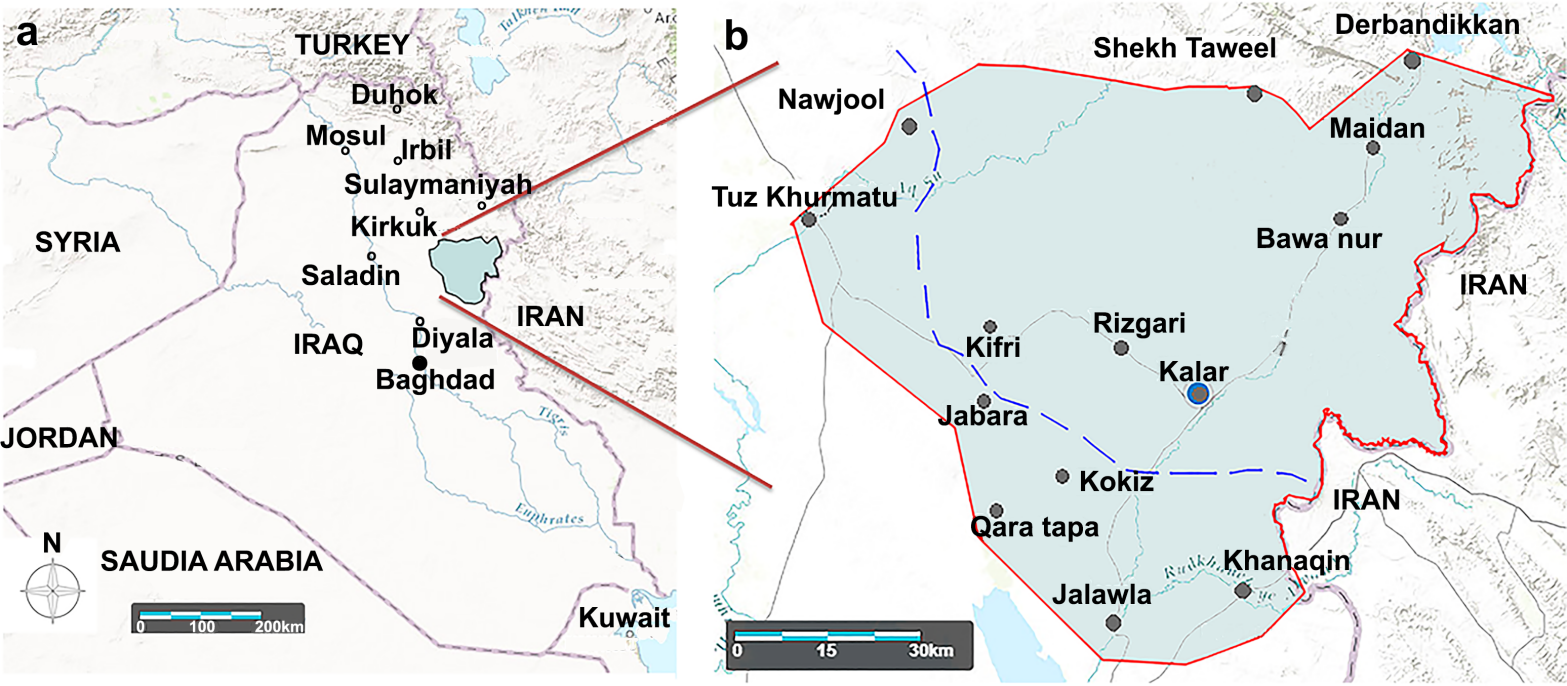
Geographical map of the study area around the Garmian region. The map was created by Landsatlook viewer (USGS Products, Data available from the U.S. Geological Survey). The interrupted blue line indicates the borderline between the north and center of Iraq by previous Iraqi governments before 2003.

Northern and central Iraq have undergone economic and humanitarian crises due to the Iraqi civil war since 2014. Moreover, the topography of both territories is different. In addition, there has been only one updated map up to 2008 based on the last report of CL incidence in Iraq by the WHO [3]. Thus, in this study, a spot map of CL cases was updated in the borderline region using Landsatlook viewer (USGS Products, Data available from the U.S. Geological Survey). The map (Fig 1a and 1b) shows the outbreak of CL from an endemic area in the Kurdistan Region of Iraq (KRI) including Diyala province to a non-endemic area inside the KRI including the Garmian administrative region.

### Epidemiological study

Data of cutaneous leishmaniasis (CL) in Iraq were collected from the WHO website [8, 15, 16], and a line graph showing annual numbers of CL cases from 1989–2015 was plotted using GraphPad Prism version 6.06 for Windows (GraphPad Software, La Jolla California USA, www.graphpad.com). Furthermore, in the area of the study (Garmian administration, Kurdistan Region, Iraq), new cases were referred by local health general practitioners to visit Kalar General Hospital in Garmian, Sulaimaniyah province to be clinically examined by dermatologists and receive Pentostam injection treatment (sodium stibogluconate). The data of clinically examined patients collected by the Department of Transmissible Diseases in Garmian from 2014–2017 were also analyzed.

### Sample preparations

Thirty samples were collected from lesions of new clinically suspected CL cases or from patients receiving early treatment in February, March and April 2017. The sample collection was performed by cleaning the skin lesions with cotton soaked in 70% ethyl alcohol and left to dry. This was followed by injecting 0.1 ml sterile normal saline into the active borders of the skin lesions using a 25-gauge insulin needle and then aspirating the fluid into sterile 1.5 ml tubes. The samples were directly preserved in 0.4 ml absolute ethanol, labeled and stored at room temperature for molecular study. Later on, the samples were submitted to the molecular laboratories of the University of Garmian which is based in the Kalar district.

### Ethics statement

Clinical samples were collected from patients who agreed to participate in this study and signed an informed consent form. The study was also approved by the Ethical Committee of the Department of Biology, College of Education, University of Garmian with permit number (85, 18/04/2017). After receiving permission from the General Directorate of Garmian Health (permit number 1550, 10/05/2017), the samples were transported to the molecular biology lab of Garmian University.

### Molecular diagnosis

A pair of primers including *Leishmania* cytochrome *b* forward (LCBF): GGTGTAGGTTTTAGTTTAGG, *Leishmania* cytochrome *b* reverse (LCBR): CTACAATATACAAATCATAATATACAATT (Macrogen Co., Seoul, KR) were exploited for amplification of the *Leishmania* cytochrome *b* gene with a product size of 866 bp as previously used for identification of almost all species of *Leishmania* by PCR and DNA sequencing [17].

Total genomic DNA of the ethanol-preserved samples was extracted by a PrimePrep Genomic DNA Extraction Kit (from tissue). The ethanol was removed from the samples by using centrifugation and washing with normal saline. The pellets were mixed with 200 μl tissue lysis buffer (TL buffer) and 20 μl proteinase K and incubated at 56°C for approximately an hour until the samples were lysed. According to the manufacturer’s instructions, DNA was isolated using ethanol and buffers then eluted with 200 μl elution buffer (TE) provided by the company (GENET BIO CO., Daejeon, KR).

Conventional PCR was performed individually for each sample in 20 μl reactions containing 1x Prime Taq premix (2x) which contains Prime Taq DNA Polymerase 1 unit, 2x reaction buffer, 4 mM MgCl_2_, enzyme stabilizer, sediment, loading dye, pH 9.0 and 0.5 mM each of dATP, dCTP, dGTP, dTTP and 0.5 μM final concentration from each of the LCBF and LCBR primers. The PCR reaction conditions were 94°C for 3 min; 40x at 94°C for 1 min, 60°C for 1 min, 72°C for 2 min; 72°C for 5 min using a thermal cycler (Mastercycler nexus, Eppendorf AG, Hamburg, Germany).

PCR products were run at 110 V for 50 min on a 1.5% agarose gel in 1x TBE (87.5 mM Tris base, 89 mM boric acid, 3 mM EDTA) and stained with Prime safe dye (GENET BIO CO., Daejeon, KR). A total of 5 μl of PCR products from nine positive samples and 5 μl (5 pmoles) of forward or reverse primers for forward or reverse sequencing, respectively, were sequenced using the Sanger method (Macrogen Co., Seoul, KR) and edited by CodonCode Aligner (CodonCode Corporation, 101 Victoria Street, Centerville, MA 02632).

### Phylogenetic study

To the best of our knowledge, no phylogenetic data were found exploring CL strains in the area along the borderline between the northern region (Kurdistan Region) and the middle part of the country based on the NCBI search engine [18] on 28/06/2017 using these keywords (Leishmaniasis Iraq PCR).

Seven high-quality sequences were submitted to the NCBI GenBank using Bankit [18]. The high-quality sequences were determined based on having high single peaks using CodonCode Aligner. The cytochrome *b* gene sequences together with those from representative strains were aligned with CLUSTAL W software and examined using the program MEGA (Molecular Evolutionary Genetics Analysis) version 7. Phylogenetic trees were constructed by the neighbor-joining method with the distance algorithms available in MEGA version 7. The distances were calculated using the Kimura 2-parameter method. Bootstrap values were determined with 1,000 replicates of the data sets [19].

## Results and discussion

### Epidemiology of CL

The CL outbreak occurred in areas between northern and central Iraq (Fig 1a). The data showed that cases of CL seemed to have originated from endemic areas like Diyala, Saladin, Mosul, and Kirkuk provinces. This outbreak may have been caused by infected people traveling from the center to the north of Iraq. It is worth mentioning that before toppling down the previous government of Iraq, i.e., before 2003, people from the southern and central provinces rarely visited Kurdistan due to strictly controlled borders (As shown in Fig 1b, interrupted blue line). After 2003, the situation changed and there was free public access into the Kurdistan Region of Iraq (KRI). Furthermore, since 2014, people have moved from the war zones of the central provinces to the Garmian region. Thus, this area can be regarded as a typical model region to understand the risk of leishmaniasis spread from endemic to non-endemic areas.

As shown on the map (Fig 1b), CL spread from endemic areas of the KRI borderline such as Jabara, Kokiz, Qara Tapa, Khanaqin, Jalawla and Tuz Khurmatu to non-endemic areas including Kalar, Kifri, and Rizgari districts was due to the arrival of refugees. This may have led to spread of the disease in other parts of Kurdistan including Sulaimaniyah, Irbil, and Duhok. Even so, temperature and humidity could be related to the distribution of the vectors and reservoirs [20]. Therefore, further study on the sandfly and animal reservoirs from both the endemic and non-endemic areas will help uncover the epidemiology of the disease. According to Alvar et al. [3], the WHO reported 1655 CL cases/ year from 2004–2008 in Iraq; the annual incidence rate was estimated from 8300 to 16,500 cases, although this number seems to be underestimated by the WHO. This could be due to a lack of diagnostic services, so the disease was not regarded as a major public health concern [21]. In addition, it is worth mentioning that not all cases were reported by the authorities for the following reasons: firstly, some infected people objected to receiving treatment due to painful intralesional injections. Secondly, some patients did not wish to visit hospitals since they use traditional medicine for treatment or believe that the lesions are self-curable. Finally, some places are far from health centers. Therefore, these data should be updated in Iraq and this will be important before introducing any CL control programs.

**Fig 2:**
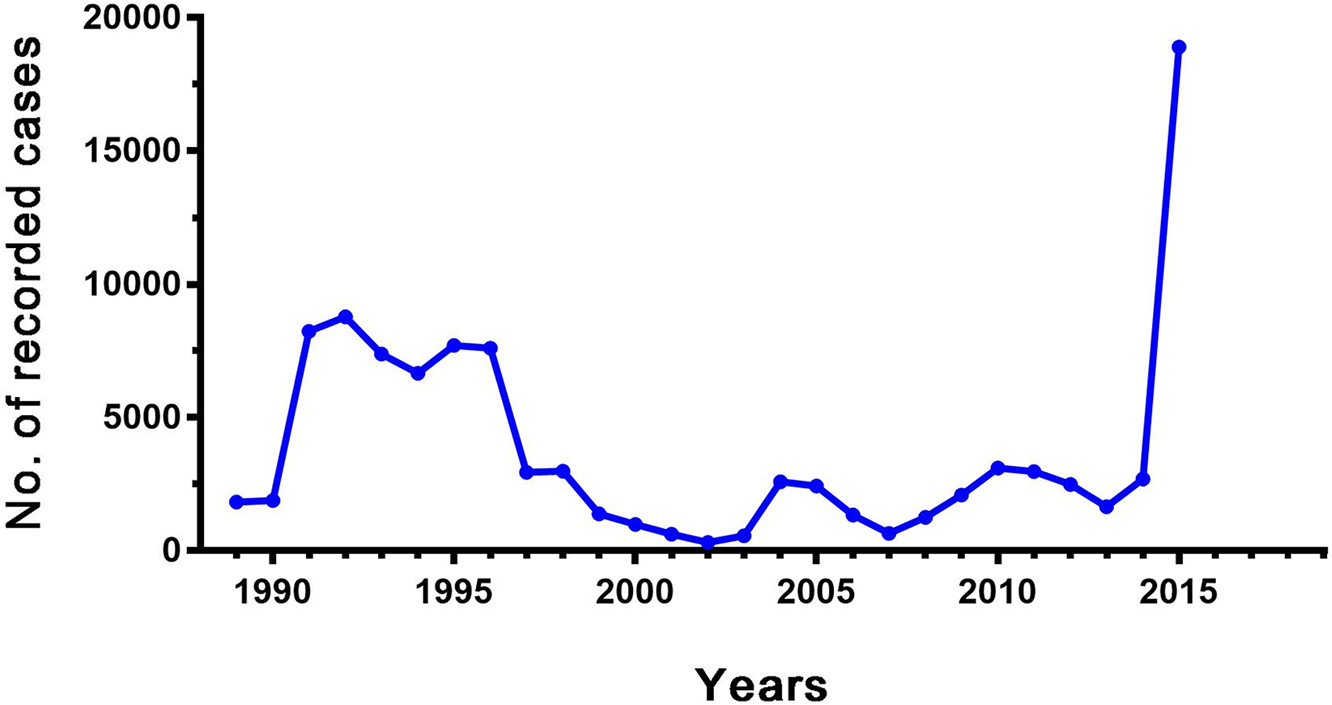
Year-wise trend of the number of reported CL cases in Iraq. Data based on WHO reports for Iraq [15, 16, 22].

In the current study, the data show the prevalence of CL in Iraq from 1989–2015 (Fig 2). As the country went through several wars, internal conflicts, economic crises, and sanctions over the previous 27 years, massive fluctuations in the number of reported CL cases can be noticed. There was a sharp increase in the number of cases after the second gulf war in 1990. This trend remained high until 1997, which could be due to discontinuation of control programs and lack of healthcare services due to the economic blockade. The number of cases remained low from 1998 until 2003, possibly due to WHO interventions [15] and control programs or relative economic growth after the removal of the sanctions in 1997. Again from 2009 the number of cases increased; however, it remained relatively low until 2014, then a sharp rise was recorded with a peak in 2015. The last increase in the number of CL cases could be due to the civil war in Iraq starting in 2014 as the war led to displacement of millions of people, especially from endemic areas to non-endemic areas, as well as a deterioration in health services.

After the introduction of malaria control programs in Iraq, the number of CL cases decreased until their discontinuation in the mid-1960s [15]. Afterwards, massive fluctuations in the number of reported cases were noticed. The number of CL cases could be related to certain factors. One of the main factors is population displacement, which brings non-immune people to endemic areas and infected people to non-endemic areas. In addition, increased contact with reservoir animals and sandfly vectors, untreated patients, malnutrition, poor sanitation and environmental changes are other possible reasons for the increase in CL cases.

**Fig 3:**
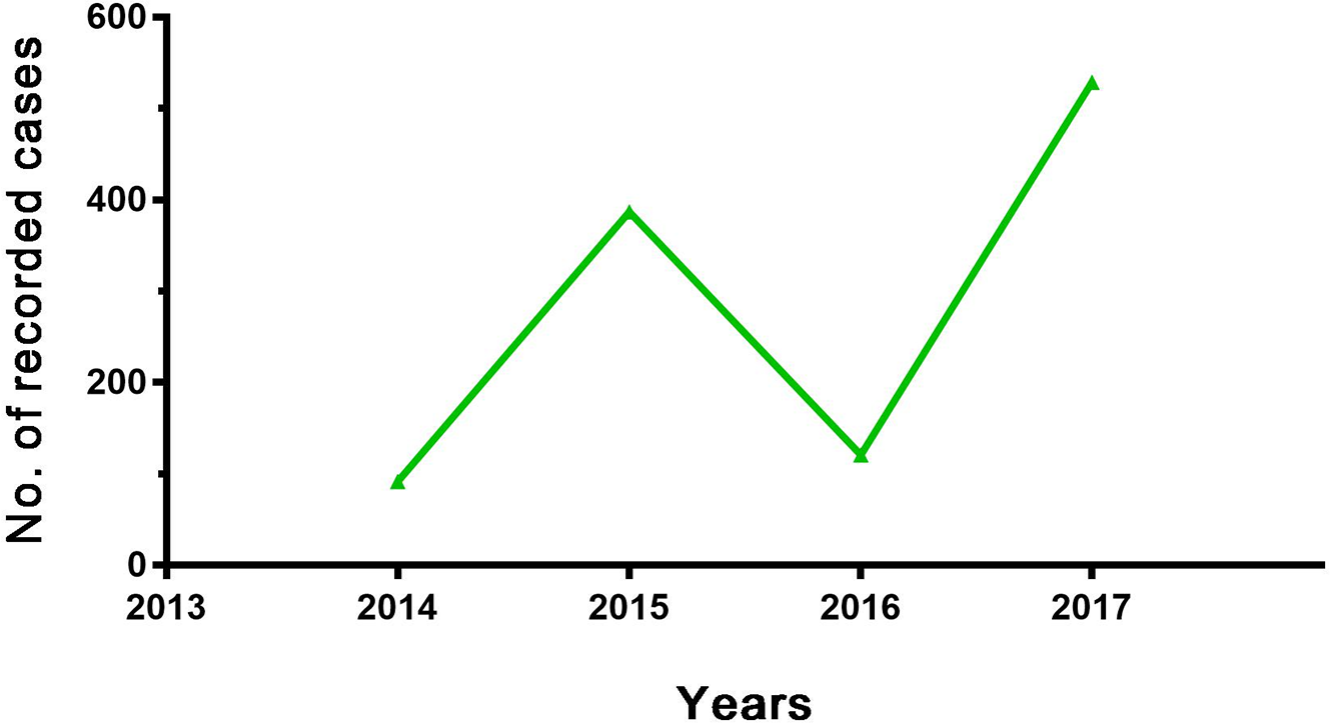
Year-wise trend of the number of reported CL cases in the Garmian region. Data were collected from reports of the Department of Transmissible Diseases of Garmian from 2014–2017.

Most of the districts of the Garmian region belong to the Sulaimaniyah governorate which is regarded as one of the non-endemic areas for CL [22]. However, after the start of the war from mid-2014, large-scale emigration of people occurred in Iraq. Garmian, as one of the border regions, housed a large number of refugees from other parts of conflict zones in Iraq; especially people from the endemic areas of Diyala, Kirkuk, Saladin and Mosul arrived in the region. This may have changed the region from a non-endemic to endemic area. This argument is supported by the large increase in the number of CL cases over the last 4 years in the region as shown in (Fig 3). Further study is required to confirm whether the region has become endemic by recording new cases who have not visited any endemic areas.

**Fig 4:**
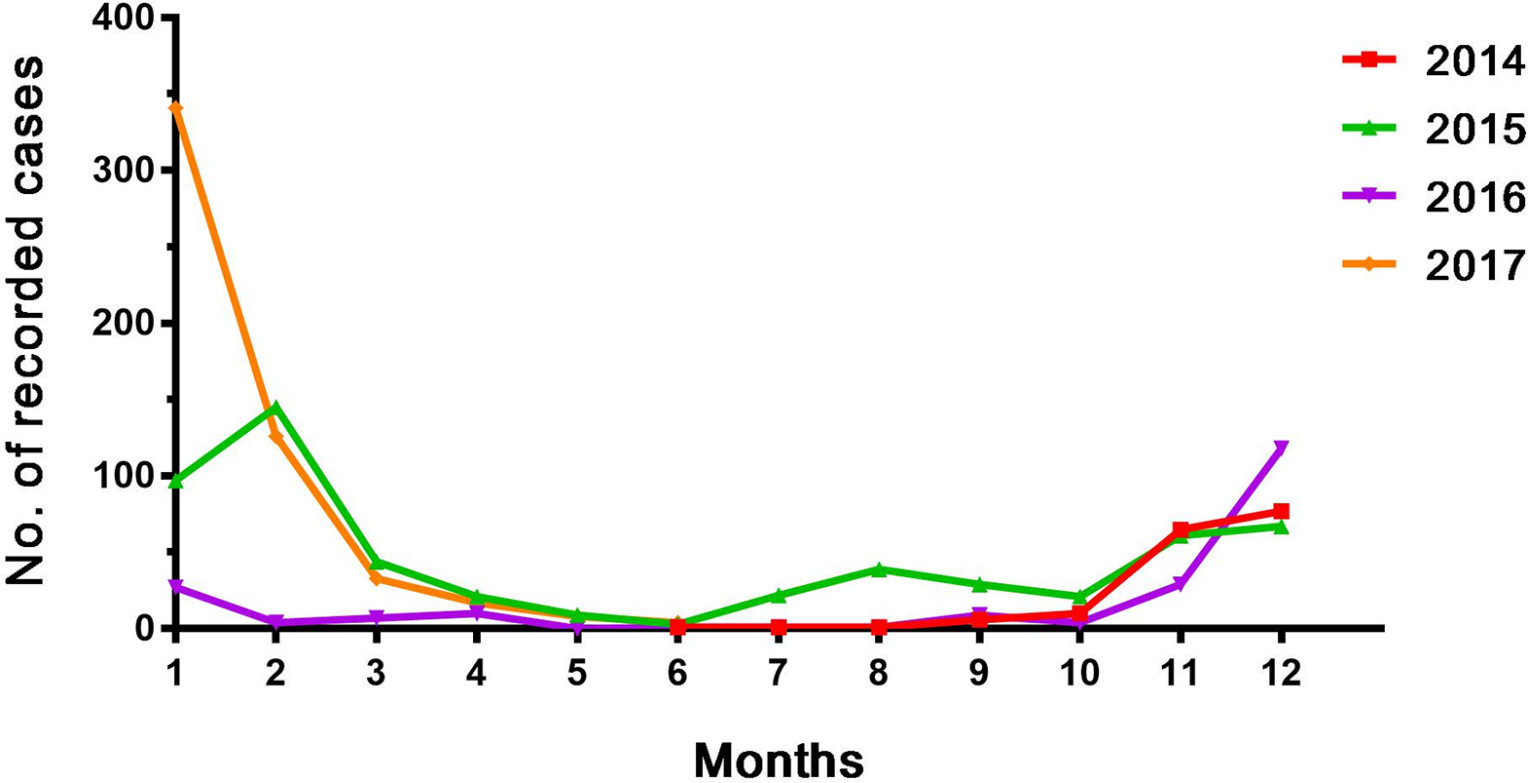
Monthly distribution of the number of reported CL cases in the Garmian region. Data were collected from reports of the Department of Transmissible Diseases of Garmian from 2014–2017.

Regarding the monthly prevalence of CL in Garmian, the number of recorded CL cases started to increase from November (Fig 4). The maximum number of CL cases was recorded in January and February. The recorded numbers decreased from March and remained low until October. These findings agreed with those reported for other parts of Iraq [10]. As the incubation period of the disease ranged from two to four months, the majority of the cases recorded during winter months were probably bitten by insects from the summer to early autumn seasons.

### Identification and genotyping of *Leishmania major* as a causative agent of CL in Iraq

**Fig 5:**
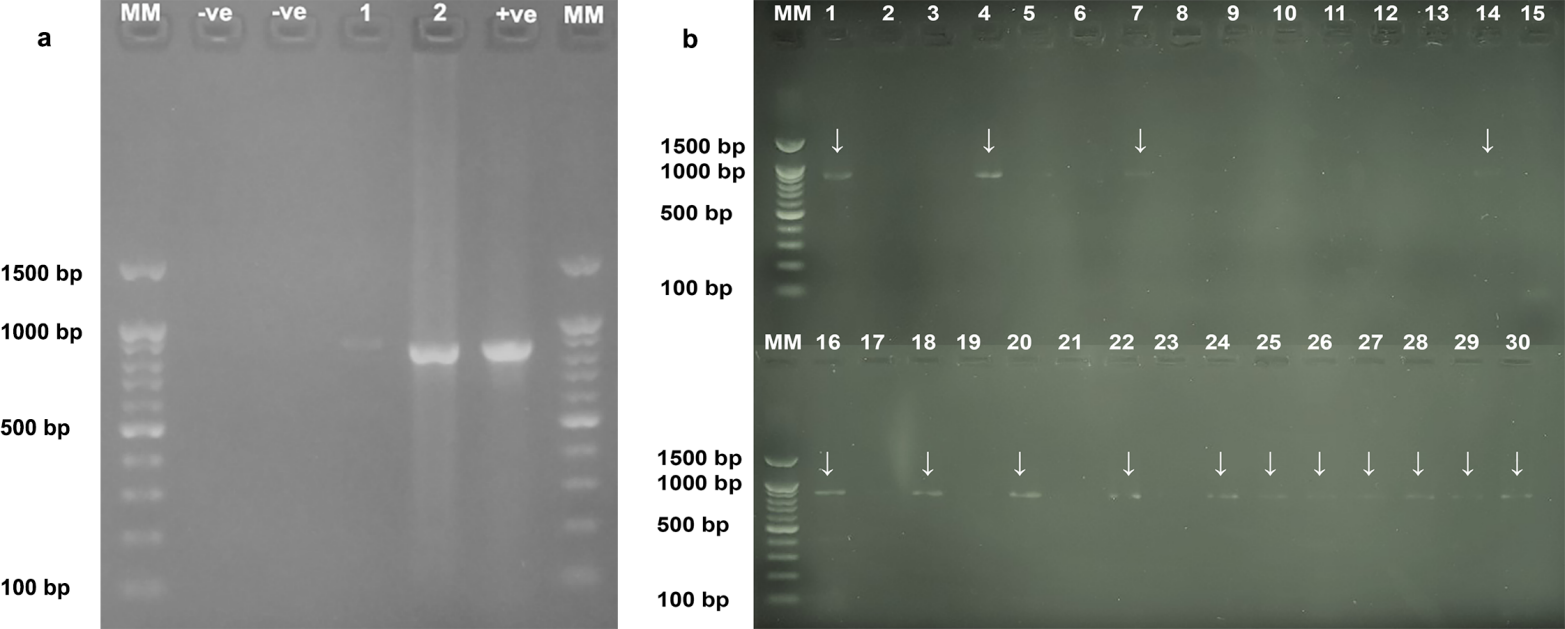
PCR products of the *L*. *major* cytochrome *b* gene run on a 1.5% agarose gel. (MM) Molecular weight marker 100–1500 bp. Panel a: -ve is negative control (without DNA); +ve is positive control (previously confirmed *L*. *major* from a dog with cutaneous lesions by sequencing [24]), sample 1 (*L*. *major* was initially confirmed from human CL by sequencing), and sample 2 (sample number 14 in Panel b. Panel b:1 to 30: skin aspirate samples; 1, 4, 7, 14, 16, 18, 20, 22, 24, 25, 26, 27, 28, 29, and 30 were positive.

Of the thirty samples collected from suspected new cases and CL patients receiving early treatment, 15 samples were positive for PCR targeting the leishmanial cytochrome *b* gene on gel electrophoresis, showing single bands with a product size of about 850–900 bp (Fig 5). Of the 15 positive samples, sequences of seven samples showing strong signals were determined, and all the *Leishmania* parasites were identified as *L*. *major* with 100% similarity with the *L*. *major* strain MRHO/IR/75/ER cytochrome *b* gene (GenBank accession number KU680828) [23]. This is the first molecular record of the *L*. *major* strain in Iraq using sequence analysis. Nonetheless, confirming only 7 cases out of a total 30 samples should be considered as a limitation of direct molecular tools for investigation of the *L*. *major* strain epidemiologically. Nucleotide sequence data reported will appear in the GenBank database under the accession numbers MF-370217-MF-370223.

**Fig 6:**
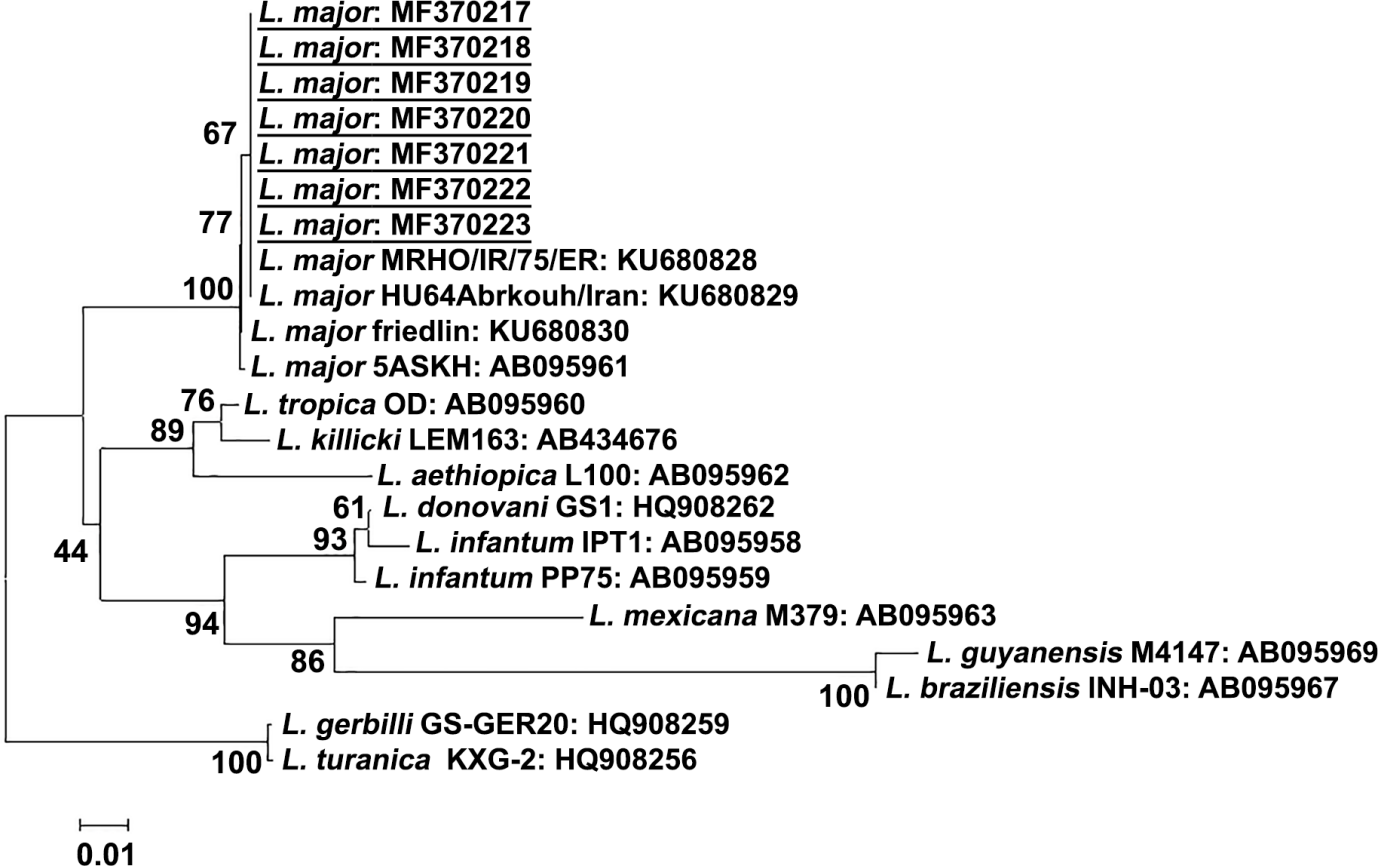
Phylogenetic analysis of cytochrome *b* gene sequences among *Leishmania* species. The scale bar represents 0.01% divergence. Bootstrap values are shown above or below branches. Underlined GenBank accession numbers represent *L*. *major* sequences identified in this study.

The cytochrome *b* gene sequences obtained in this study were subjected to phylogenetic analysis together with those from representative *Leishmania* strains. The phylogenetic data indicated that *Leishmania* strains in the borderline area between northern and central Iraq were closely related to the Iranian MRHO/IR/75/ER strain and other *L*. *major* strains of Old World CL (Fig 6). This result is not surprising as the region shares its border with Iran which is the nearest country to the endemic area of central Iraq. Particularly, Diyala province has had a commercial relationship with Iran through the border close to the Khanaqin district since 2003.

Microsatellite analyses using specimens from geographically isolated areas, Central Asia, the Middle East and Africa showed *L*. *major* has little genetic variation when compared to *L*. *tropica* [25, 26]. This may partly explain the slight variation in *L*. *major* identified in this study. Further large-scale genetic analysis using more sensitive methods such as microsatellite analysis will be necessary in the future.

The exact origin of the parasite is unknown although there has been a history of the disease in Iraq [27]. In addition, there has been a long history of pilgrims visiting from Iran to sacred shrines in central and southern Iraq via either the Garmian region or central provinces.

The reservoirs of *L*. *major* have not been identified in Iraq, but in Iran, four gerbil species were identified as main reservoirs including *Rhombomys opimus*, *Meriones libycus*, *Meriones hurrianae* and *Tatera indica* [28]. Nevertheless, we recently identified the *L*. *major* MRHO/IR/75/ER strain from cutaneous lesions of a dog in the study area [24].

Further studies will reveal the transmission vectors and reservoirs and aid in control of the outbreaks. To our knowledge, this is the first record of a phylogenetic study in Iraq concerning *L*. *major* causing CL prevalence. The findings are also significant for future creation of vaccines against the *Leishmania* strain in Iraq and it is important to understand the global prevalence and epidemiology of the *Leishmania* strains.

In conclusion, we identified the *L*. *major* strain in Iraq for the first time using PCR and DNA sequencing. In addition, phylogenetic study revealed the main *Leishmania* genotype causing health problems in the borderline area between the non- endemic area in the north and the endemic region of central Iraq. The identified parasite was similar to the MRHO/IR/75/ER strain which is endemic in Iran. Furthermore, we reported new CL cases in the borderline area between central and northern Iraq, particularly in the Garmian region. This region can be regarded as a model for further study of epidemic CL outbreaks to other non-endemic areas. This study also suggests that researchers conduct more studies regarding the threat of CL which may spread globally to countries where both reservoirs and sandflies are present.
